# Influence of High-Power Laser Cleaning on Oxide Layer Formation on 304L Stainless Steel

**DOI:** 10.3390/mi16121366

**Published:** 2025-11-30

**Authors:** Hyun Jong Yoo, HyeonSik Kang, Youngki Kim, Changkyoo Park

**Affiliations:** 1 Department of Laser and Electron Beam Technologies, Korea Institute of Machinery and Materials, Daejeon 34103, Republic of Korea; 2 Department of Materials Science and Engineering, Hanbat National University, Daejeon 34158, Republic of Korea; 3 Department of Materials Science and Engineering, Seoul National University of Science and Technology, Seoul 01811, Republic of Korea; 4 Department of Reliability, Virtual Engineering Platform Research Division, Korea Institute of Machinery and Materials, Daejeon 34103, Republic of Korea

**Keywords:** high-power laser, laser surface cleaning, oxide formation, SS304L

## Abstract

In this study, a kW-level high-power Nd:YAG nanosecond laser was adopted to eliminate a corrosion layer on a 304L stainless steel (SS304L) surface. Four different laser cleaning (LC) processes with various hatch distances and loop counts were adopted. The energy-dispersive X-ray spectroscopy (EDX) analysis revealed that the corrosion layer was successfully eliminated via the LC process. However, the electron probe X-ray microanalysis (EPMA) analysis confirmed that a Cr-based oxide layer with a thickness of a few micrometers had developed on the surface of SS304L by the LC process. Moreover, Cr-depleted regions were generated in the subsurface owing to the Cr consumption for oxide layer development. The surface temperature during the LC process strongly affected the thickness of oxide layers. The oxide layer and Cr-depletion formation can affect the subsequent manufacturing processes, including welding and molding. Moreover, those can influence the materials’ properties themselves, where the laser-cleaned workpieces may be used. Therefore, it is critical to characterize the relation between LC process parameters and microstructural alteration.

## 1. Introduction

Grade 304L stainless steel (SS304L) contains 18–20% of Cr and 8–10.5% of Ni. This austenitic stainless steel has excellent mechanical properties and corrosion resistance, making it suitable for applications in pressure vessels, automobiles, nuclear plants, ships, and heat exchangers [1,2,3,4,5,6,7]. The excellent corrosion resistance of SS304L originates from the existence of a surface Cr-based oxide as a passivation layer, which prevents further oxidation. However, when SS304L is subjected to temperatures between 425 and 870 °C, Cr carbides develop along the grain boundary at the subsurface [8,9]. Cr-depleted regions were generated near the grain boundaries, resulting in susceptibility to intergranular corrosion and cracking. This deterioration, known as sensitization, tends to happen in parts exposed to elevated operating temperatures, such as power plants, automotive engine systems, and heat exchangers [10,11]. Therefore, regular surface maintenance is critical to eliminate surface corrosion and avoid unexpected corrosion-induced failures.

Conventional surface maintenance techniques include abrasive blasting and chemical etching. Abrasive blasting uses abrasive materials (e.g., metal balls and sand) with high kinetic energy, and discharges abrasive materials against the workpiece to remove the surface contaminants [12,13,14,15]. Chemical etching uses strong acid solutions, such as NH_4_CO_3_, HF, and ethylene diamine tetra acetic acid, to etch away the surface contaminants [16]. However, these techniques have obvious inherent limitations. Abrasive blasting may generate surface defects and plastic deformation because the highly propelled abrasive materials collide with the workpiece surfaces [17,18,19]. In addition, airborne abrasive particles and dispersed small contaminants can lead to environmental concerns and pose respiratory health risks for workers [20,21], whereas chemical etching can induce surface discoloration and produce substantial volumes of toxic acid waste. This can cause environmental contamination; therefore, users should invest money to install a purification system [22,23].

Laser cleaning (LC) is a potential technique for removing surface residues before its characteristics of easy control, non-contact, and high precision and efficiency [24,25,26]. Moreover, LC is considered an eco-friendly technique because it does not generate any additional wastes, including acidic solutions and spent abrasive materials [27]. Roberts et al. used a tens of Watts Nd:YAG laser and succeeded in achieving radioactive decontamination of stainless steel [28]. Shamsujjoha et al. conducted laser surface cleaning of the passivation layer on high-strength steel and investigated its effect on surface roughness, residual stress, and fatigue [29,30]. Yoo et al. removed paint and corrosion layers on SS304L with a 1.2 kW Nd:YAG laser and investigated the mechanical properties, as well as wear and electrochemical behavior [31]. Laser surface cleaning removes the surface contaminations based on the laser ablation phenomenon, which refers to the conversion of solid-state materials into plasma with a high laser energy density [32,33]. However, during the LC process, the materials absorb laser energy, increasing the surface temperature and oxide layer formation. D.P. Adams et al. investigated the formation of Cr-based oxides on SS304L during laser color marking [34]. S.K. Lawrence et al. studied the laser-induced Cr-based oxide formation and its influences on the corrosion behaviors of SS304L [35].

However, as far as we know, the influence of the kW level high-power nanosecond laser-based LC process on the corrosion layer elimination of stainless steel, as well as its effects on the surface oxide layer formation, has rarely been investigated. The high-power LC process may have increased the surface temperature of SS304L. This contributes to the development of a Cr-based oxide layer on the surface, and the Cr-depleted regions can develop adjacent to the oxide layer. Eventually, this microstructural modification can alter the mechanical properties and corrosion behaviors after the LC process. This phenomenon should be prevented because the laser-cleaned parts must be reused. In this study, a 1.2 kW-level nanosecond laser was adopted to eliminate the corrosion layer on SS304L. Artificially corroded SS304L specimens were prepared, and the LC process was performed with different laser cleaning strategies to investigate the relation between the oxide layer formation and laser cleaning parameters. The scanning electron microscopy (SEM) and electron probe X-ray microanalyzer (EPMA) analyses were conducted to analyze the development of a Cr-based oxide layer on the SS304L surface after the LC process.

## 2. Experimental Set-Up

### 2.1. Materials and Laser Set-Up

In this study, commercial cold-rolled SS304L (POSCO, Pohang, Rebulic of Korea) was utilized, and Table 1 shows the chemical composition. The SS304L specimens with dimensions of 100 (W) × 100 (L) × 10 (H) mm^3^ underwent annealing at 1050 °C for 90 min, followed by cooling to 25 °C in an Ar atmosphere. The corroded SS304L specimens (denoted as corrosion) were prepared in a 500 °C muffle furnace (FP-03, WiseTherm, Wonju, Rebulic of Korea) with a continuous spray of 20% NaCl solution (Sigma-Aldrich, St. Louis, MO, USA) on SS304L every 12 h.

**Figure 1:**
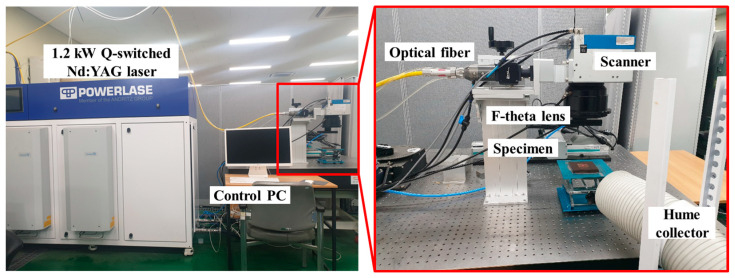
1.2 kW-level nanosecond laser set-up for the SS304L laser cleaning process.

The corrosion layer on SS304L was laser-cleaned using a 1.2 kW-level nanosecond laser (Rigel i1200, PowerLase, Crawley, UK), as shown in Figure 1. The laser power was 1140 W with a pulse duration of 54.4 ns, pulse frequency of 8 kHz, and central wavelength of 1064 nm. The laser beam size of 2.1 mm was examined using a charge-coupled device camera (Ophir Photonics, MA, USA). The laser beam quality factors (M^2^) were 29.7 in the horizontal (x) direction and 30.2 in the vertical (y) direction. Consequently, the corresponding laser energy density was 4.11 J/cm^2^, calculated by the following equation:

$$Laser\;energy\;density\;\left(J/{cm}^{2}\right)=\;\frac{Laser\;Power\;(W)}{Repetition\;rate\;\left(Hz\right)\times Laser\;beam\;({cm}^{2})}$$

**Figure 2:**
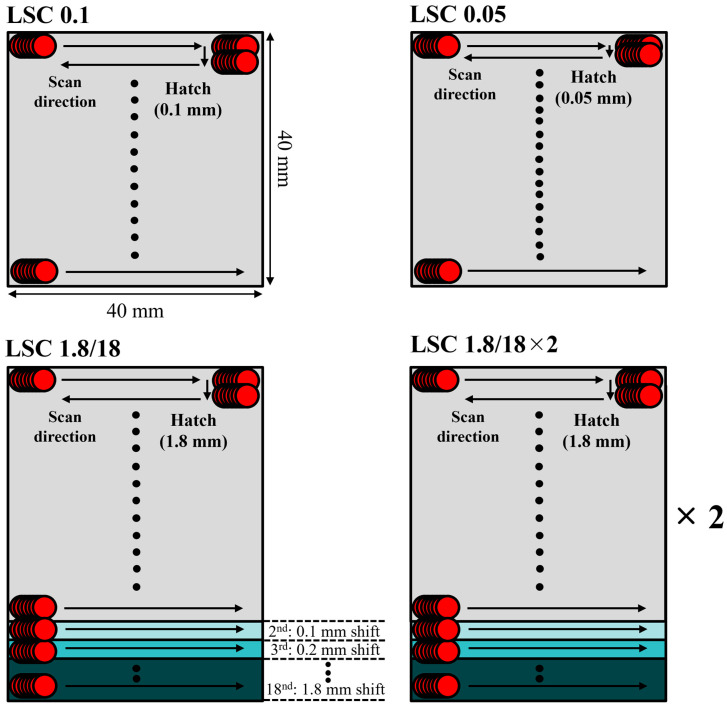
Four different laser cleaning strategies for the SS304L cleaning. Red circles represent a laser beam, and black dots represent an omission of the laser beam scan.

Schematic illustrations for the LC processes are shown in Figure 2. The dimensions of the LC process were 40 × 40 mm^2^. The LC process employed three hatch distances (0.05, 0.1, and 1.8 mm) with a fixed scanning speed of 1000 mm/s. A two-dimensional galvanometer scanner (SUPERSCAN IIE-30, Raylase, Weßling, Germany) was adopted for the scanning of the laser beam. A single laser cleaning process was conducted for hatch distances of 0.05 and 0.1 mm (denoted as LC 0.05 and LC 0.1, respectively). In the case of a hatch distance of 1.8 mm, the beginning position of the LC process was shifted downwards by 0.1 mm for each following scan, and the LC process was repeated eighteen times to fulfill the equivalent beam path distance with that of the LC 0.1 (denoted as LC 1.8/18). The specimen was denoted as the LC 1.8/18×2 with two repetitions of the LC 1.8/18 process. These LC processes were adopted in this study based on our previous studies, which already verified a perfect corrosion layer removal [31,36,37]. A two-color pyrometer monitored the surface temperatures at the center of the LC region during the LC process.

### 2.2. Microstructure

The specimens (LC 0.05, LC 0.1, LC 1.8/18, and LC 1.8/18×2) were sectioned to dimensions of 1 × 1 × 1 cm^3^ using a low-speed cutting wheel at the center of the LC region for microstructural characterization. Cross- and top-sectional microstructural characterizations were performed using optical microscopy (OM; KH-8700, HIROX, Tokyo, Japan) and SEM (SNE-4500, SEC, Suwon, Rebulic of Korea). The chemical compositions before and after the LC process were characterized using energy-dispersive X-ray spectroscopy (EDX) and EPMA (EPMA-1610, Shimadzu, Kyoto, Japan). The chemical composition was characterized to analyze the laser-cleaning effectiveness and oxide development on the surface after the LC process.

## 3. Results and Discussion

### 3.1. Laser Cleaning Process

**Figure 3:**
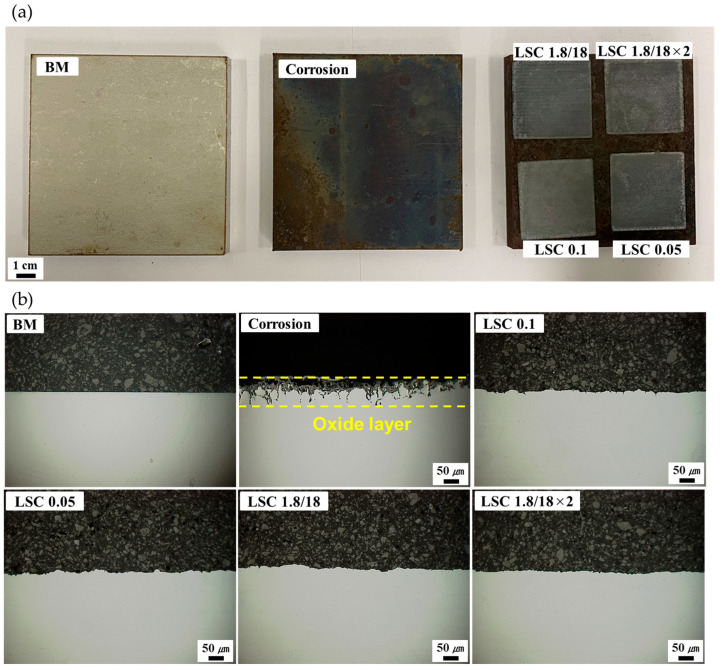
(**a**) Specimen images of the BM, corrosion, and laser-cleaned specimens. (**b**) Cross-sectional OM images of the BM, corrosion, LC 0.1, LC 0.05, LC 1.8/18, and LC 1.8/18×2.

Figure 3a shows the images of the base metal (BM), corrosion, and laser-cleaned specimens. A reflective metal surface was examined for the BM, while dark brown corrosion products were observed on the surface for the corrosion. After the LC process, the bright metal surfaces were again exposed, indicating that the corrosion layer was successfully eliminated via the LC process. Figure 3b shows the cross-sectional OM images of the BM, corrosion, and laser-cleaned specimens. Approximately 100 μm thickness of corrosion layer was examined; however, no corrosion layer was observed in the laser-surface-cleaned specimens.

**Figure 4:**
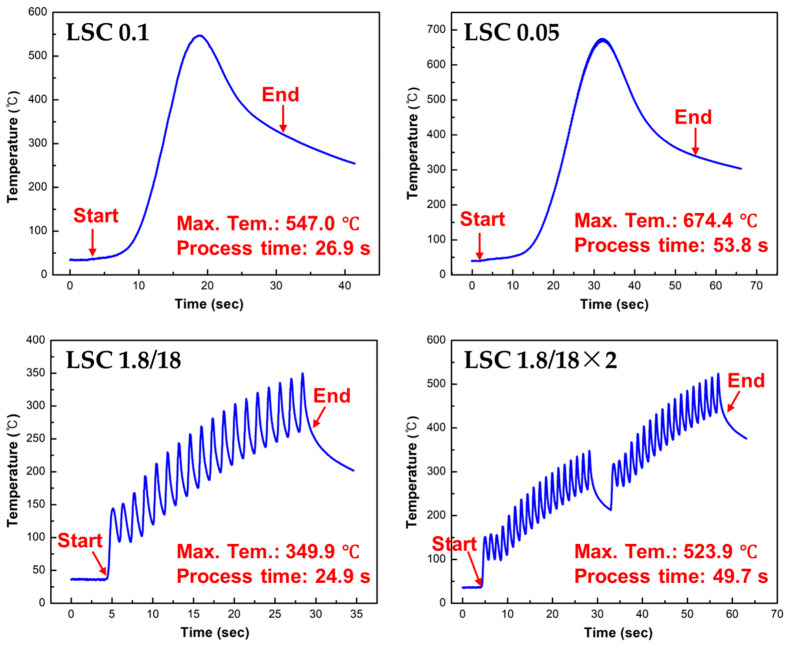
Measured surface temperature during laser surface cleaning process.

To investigate the difference in the oxide layer thickness, the surface temperature at the center of the laser-cleaned region was monitored during the LC process (Figure 4). For the LC 0.1, the LC process time was 26.9 s, and the maximum temperature was measured as 547.0 °C. On the other hand, a higher maximum surface temperature (674.7 °C) was measured for the LC 0.05 with the LC process time of 53.8 s. A smaller hatch distance of 0.05 mm caused a larger LC process time, resulting in a higher laser energy input and surface temperature on the specimen during the LC process. Meanwhile, relatively lower surface temperatures were found to be 349.9 and 523.9 °C for the LC 1.8/18 (process time: 24.9 s) and LC 1.8/18×2 (process time: 49.7 s), respectively. Repetitive fluctuations in the measured surface temperature profile were observed for the LC 1.8/18 and LC 1.8/18×2, owing to the multiple loop counts of the LC process with a large hatch distance (i.e., 1.8 mm). This result indicates that the one-time LC process with a small hatch distance induced heat accumulation during the LC process; therefore, the surface temperature gradually increased and reached its maximum surface temperature. By contrast, multiple loop counts of the LC process with a large hatch distance prevented heat accumulation. Consequently, a lower surface temperature was obtained for the LC 1.8/18 than that of the LC 0.1, although the two specimens had equivalent laser beam paths and energy inputs. For the LC 1.8/18×2, the double laser beam paths and energy input were applied to the specimen compared with those of the LC 0.1, while a lower maximum surface temperature was obtained.

### 3.2. Oxide Layer Formation

**Figure 5:**
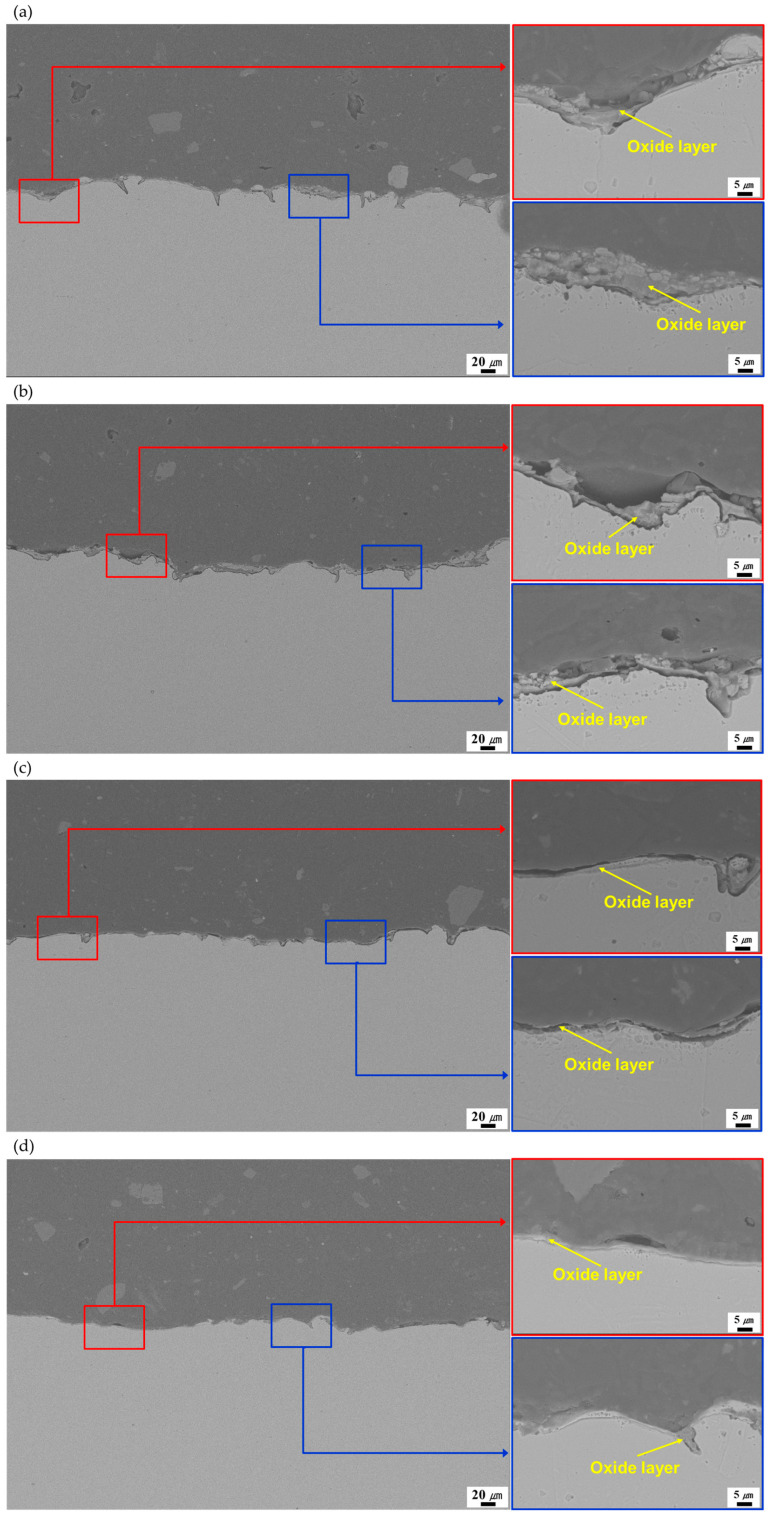
Cross-sectional low magnification SEM image (left) and high magnification SEM images (right) for the (**a**) LC 0.1, (**b**) LC 0.05, (**c**) LC 1.8/18, and (**d**) LC 1.8/18×2.

Figure 5 shows the SEM images of the LC 0.1, LC 0.05, LC 1.8/18, and LC 1.8/18×2. High-magnification SEM images were obtained at the red and blue boxes in the low-magnification SEM image. After LC, each laser-cleaned specimen had an oxide layer on its surface. Relatively larger thickness oxide layers were developed for the LC 0.1 and LC 0.05 compared with those of the LC 1.8/18 and LC 1.8/18×2. The smallest oxide layer thickness was obtained for LC 1.8/18, which exhibited the lowest surface temperature with the shortest LC process time. The LC 1.8/18×2 and LC 0.1 had intermediate oxide layer thicknesses owing to intermediate surface temperatures during the LC process. The largest oxide layer thickness was obtained for the LC 0.05. This was because the oxidation may be easily proceeded during the LC process for the LC 0.05 owing to the highest surface temperature (i.e., 674.4 °C) with the longest LC process time.

**Figure 6:**
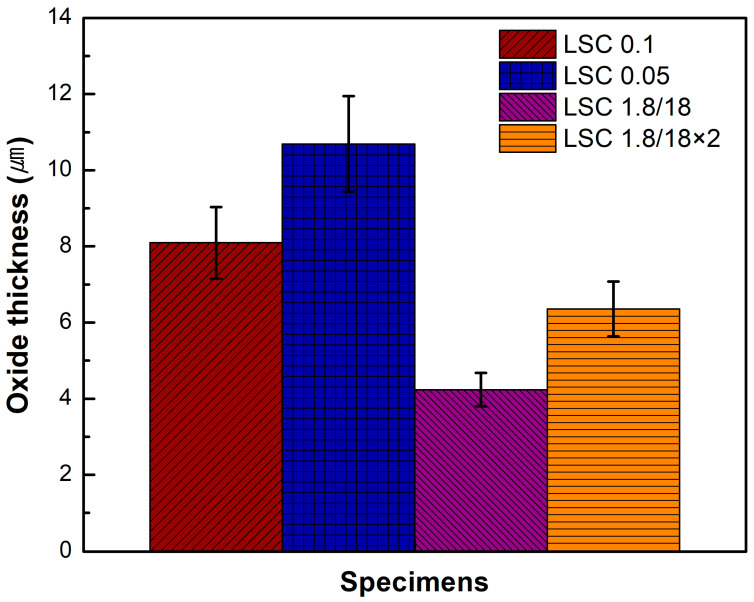
Measured oxide layer thickness on the laser-cleaned surface after the LC process.

The oxide layer thickness of the laser-cleaned specimens was measured at fifteen different locations using the SEM images, and the average values of the oxide layer thickness are presented in Figure 6. For the LC 0.1 and LC 0.05, the measured oxide layer thicknesses were 8.1 and 10.7 μm, respectively; these values decreased to 4.2 and 6.4 μm for the LC 1.8/18 and LC 1.8/18×2, respectively. The smallest oxide layer thickness was obtained for the LC 1.8/18 owing to the lowest surface temperature and shortest LC process time during the LC process.

### 3.3. Development of Cr-Depleted Regions

**Figure 7:**
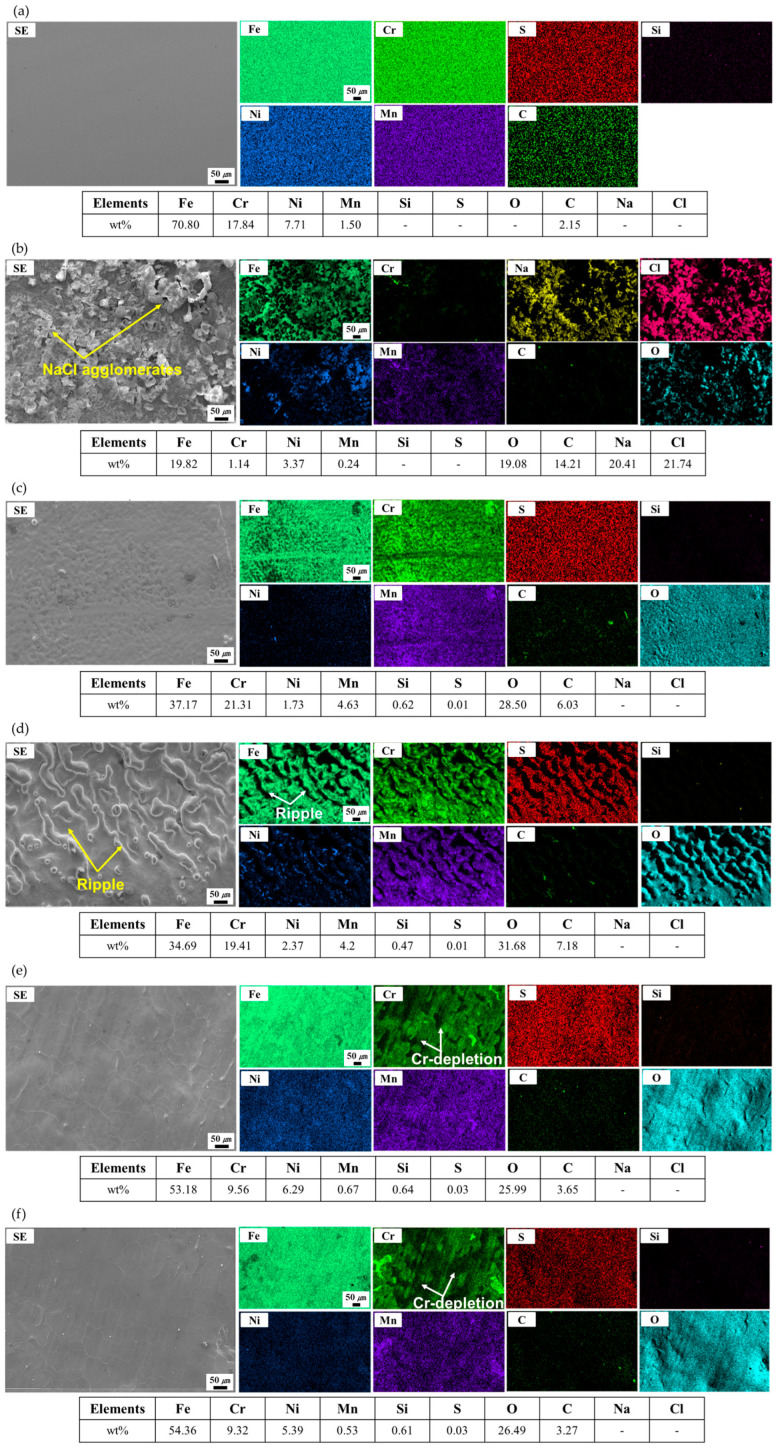
Top-sectional SEM images and corresponding elemental distribution maps and chemical compositions obtained from the EDX analysis for the (**a**) BM, (**b**) corrosion, (**c**) LC 0.1, (**d**) LC 0.05, (**e**) LC 1.8/18, and (**f**) LC 1.8/18×2.

The EDX characterization was conducted to examine the corrosion removal level after the LC process. Figure 7 shows a top-sectional SEM image and its corresponding elemental distribution maps and chemical compositions for the BM, corrosion, and laser-cleaned specimens. For the BM, the homogeneous elemental distributions were characterized, as shown in Figure 7a. Moreover, a similar chemical composition was examined by the EDX analysis to that of SS304L in Table 1. In contrast, segregated elemental distributions were observed for corrosion (Figure 7b). A high weight percentage of O was detected because of the corrosion layer produced on the surface. Moreover, Na and Cl were widely dispersed on the surface since the NaCl solution was utilized to induce artificial corrosion in SS304L. Figure 7c,d shows the laser cleaning result for the LC 0.1 and LC 0.05, respectively. Relatively homogeneous elemental distributions were examined and compared with those of the corrosion. Moreover, Na or Cl was not detected for the LC 0.1 and LC 0.05. This outcome suggests that the LC processes successfully eliminated the corrosion layer. However, significantly high weight percentages of O and Cr were examined for the LC 0.1 and LC 0.05. The weight percentages of O and Cr were 28.5 and 21.31% for the LC 0.1 and 31.68 and 19.41% for the LC 0.05. This implies that the Fe- and Cr-based oxide layer developed after the LC process. Moreover, for the LC 0.05, micro-sized ripples were produced at the surface. This was because the subsurface was slightly melted and re-solidified during the LC process owing to a high surface temperature. As a result, non-uniform Fe and Cr elemental distributions were observed. The LC 1.8/18 and LC 1.8/18×2 (Figure 7e,f) also show relatively homogeneous elemental distributions with no detection of Na or Cl elements, revealing a perfect laser cleaning of the corrosion layer. Compared with the LC 0.1 and LC 0.05, lower weight percentages of O and Cr were observed; however, a high weight percentage of Fe was also observed for the LC 1.8/18 and LC 1.8/18×2, revealing the Fe- or Cr-based oxide layer development at the surface. Moreover, a non-uniform Cr elemental distribution was detected with multiple Cr-depleted regions for the LC 1.8/18 and LC 1.8/18×2.

**Figure 8:**
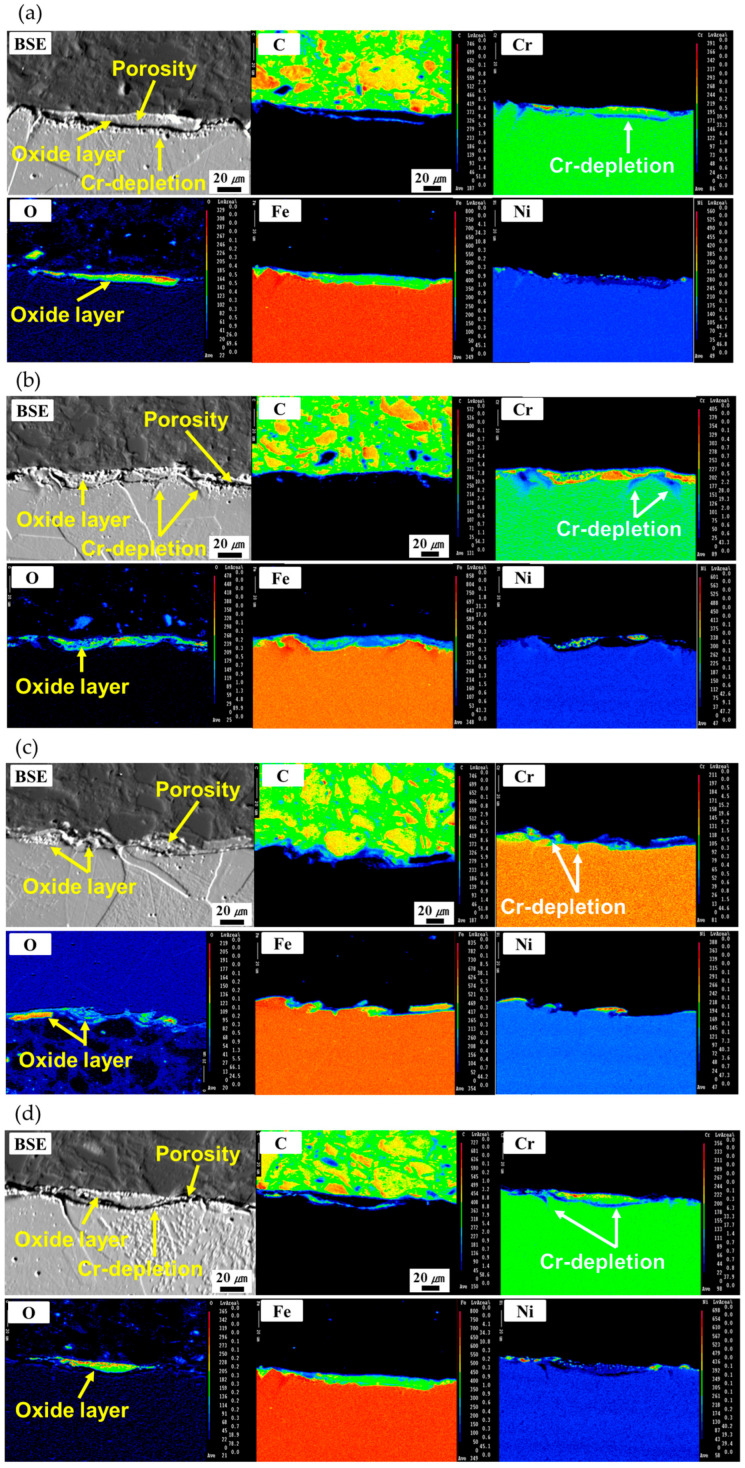
BSE image and corresponding elemental distribution maps by the EMPA analysis for the (**a**) LC 0.1, (**b**) LC 0.05, (**c**) LC 1.8/18, and (**d**) LC 1.8/18×2.

The EPMA analysis was performed to characterize the developed oxide layer and Cr-depletion after the LC process. Figure 8 shows the cross-sectional backscattered electron (BSE) image and its corresponding elemental distribution maps of C, Cr, O, Fe, and Ni for the LC 0.1, LC 0.05, LC 1.8/18, and LC 1.8/18×2. For the LC 0.1 and LC 0.05 (Figure 8a,b), approximately 10 μm thickness of oxide layer was generated after the LC process. Relatively high amounts of Cr and O and low amounts of iron were detected on the surface compared with those in the deep regions of the specimen. This result reveals the development of a Cr-based oxide layer at the surface after the LC process, substantiating the high amount of Cr at the surface in the EDX analysis (Figure 7). Because of the development of the Cr-based oxide layer, a large amount of Cr, adjacent to the Cr-based oxide layer, was consumed. As a result, the Cr-depleted regions were generated near the oxide layer with multiple pores. For the LC 1.8/18 and LC 1.8/18×2 (Figure 8c,d), the relatively smaller thickness of Cr-based oxide layers (approximately 5 μm thickness) was developed compared with those of the LC 0.1 and LC 0.05. A relatively larger oxide layer thickness was observed for the LC 1.8/18×2 than that of the LC 1.8/18. Moreover, only a marginal Cr-depleted region was created for the LC 1.8/18, whereas the LC 1.8/18×2 showed a relatively large Cr-depleted region (a Cr-depleted region comparable to that of the LC 0.1). This was because the LC 1.8/18×2 showed a higher surface temperature and longer process time during the LC process compared with the LC process. The EDX and EPMA results reveal that the formation of oxide layer and Cr-depleted regions during the LC process were strongly dependent on the laser cleaning parameters because those determined the surface temperature and process time during the LC process.

## 4. Conclusions

In this study, a laser surface cleaning process was conducted to eliminate the corrosion layer on SS304L. Various hatch distances and loop counts were adopted for four different LC processes; LC 0.1 (hatch distance: 0.1 mm; loop count: 1), LC 0.05 (hatch distance: 0.05 mm; loop count: 1), LC 1.8/18 (hatch distance: 1.8 mm; loop counts: 18), and LC 1.8/18×2 (hatch distance: 1.8 mm; loop counts: 36). The EDX analysis revealed that every laser-cleaned specimen exhibited perfect corrosion layer removal on the SS304L surface. However, the EDX and EPMA analyses confirmed that the Cr-based oxide layer was created on the SS304L surface after the LC process. Moreover, the Cr-depleted regions developed adjacent to the oxide layer because Cr was consumed during the formation of the oxide layer. The thickness of the oxide layer was strongly dependent on the LC process because parameters owing to different surface temperatures and process time. The smallest oxide layer thickness of 4.2 μm was obtained for the LC 1.8/18 owing to 341.7 °C and shortest process time (24.9 s) during the LC process. By contrast, the LC 0.05 showed the largest oxide layer thickness (10.7 μm) with the highest surface temperature of 674.4 °C and the longest process time of 53.8 s during the LC process. Notably, a relatively thick oxide layer can develop after the high-power LC process with a longer process time and higher surface temperature. The formation of an oxide layer can affect the subsequent manufacturing processes (e.g., welding and molding) where the laser-cleaned workpieces may be used. Moreover, the development of Cr-depleted regions and porosity in the microstructure can influence the wear resistance and mechanical properties of the surface. Such changes become particularly critical when the LC-treated surface is intended for applications requiring high surface integrity. For example, the automobile press molds often demand a good surface finish (~1 μm) and high surface hardness (~600 HV) to maintain forming quality and stability. Due to the excessive oxide growth or Cr-depletion generated during the LC process, the laser-cleaned mold may fail to meet the required specification, which may deteriorate mold performance and service life. Therefore, minimizing the oxide layer and Cr-depletion formation by optimizing the LC process parameters is critical for a kW-level high-power LC process.

## Figures and Tables

**Table 1:** Chemical composition of SS304L (POSCO).

**Elements**	**Cr**	**Ni**	**Mn**	**Si**	**Cu**
wt%	18.14	8.604	1.462	0.386	0.216
**Elements**	**Mo**	**P**	**C**	**S**	**Fe**
wt%	0.112	0.031	0.021	0.002	71.026

## Data Availability

The data presented in this study are available on request from the corresponding author. Large and complex datasets are required significant effort to properly format and document for public use.

## Abbreviations

SS304L	304L stainless steel
LC	Laser cleaning
EDX	Energy-dispersive X-ray spectroscopy
EPMA	Electron probe X-ray microanalysis
SEM	Scanning electron microscopy
OM	Optical microscopy
BM	Base metal
BSE	Backscattered electron
